# Supplementary material for: SyNDI: synchronous network data integration framework
Source: BMC Bioinformatics. 2018 Nov 6;19:403. doi: 10.1186/s12859-018-2426-5 (PMC6219086; doi:10.1186/s12859-018-2426-5)
Supplement: Supplementary file 5 — Files of the example presented in Additional file 4. This additional file is a zip package that contains all files used in Additional file 4. Detailed descriptions of each of these files are presented in a README file included in this zip package. (GZ 30411 kb) [file 12859_2018_2426_MOESM5_ESM.gz › SyNDITools/Meme2Fimo/meme_4.9.0_4.tar.gz!/meme_4.9.0/doc/meme.html]

MEME — Multiple EM for Motif Elicitation


# MEME — Multiple EM for Motif Elicitation

## USAGE:

meme <dataset> [optional arguments] \| <dataset> \| file containing sequences in FASTA format; \| [-h] \| print this message; \| [-o <output dir>] \| name of directory for output files will not replace existing directory; \| [-oc <output dir>] \| name of directory for output files will replace existing directory; \| [-text] \| output in text format (default is HTML); \| [-dna] \| sequences use DNA alphabet; \| [-protein] \| sequences use protein alphabet; \| [-mod oops\|zoops\|anr] \| distribution of motifs; \| [-nmotifs <nmotifs>] \| maximum number of motifs to find; \| [-evt <ev>] \| stop if motif E-value greater than <evt>; \| [-nsites <sites>] \| number of sites for each motif; \| [-minsites <minsites>] \| minimum number of sites for each motif; \| [-maxsites <maxsites>] \| maximum number of sites for each motif; \| [-wnsites <wnsites>] \| weight on expected number of sites; \| [-w <w>] \| motif width; \| [-minw <minw>] \| minumum motif width; \| [-maxw <maxw>] \| maximum motif width; \| [-nomatrim] \| do not adjust motif width using multiple alignments; \| [-wg <wg>] \| gap opening cost for multiple alignments; \| [-ws <ws>] \| gap extension cost for multiple alignments; \| [-noendgaps] \| do not count end gaps in multiple alignments; \| [-bfile <bfile>] \| name of background Markov model file; \| [-revcomp] \| allow sites on + or - DNA strands; \| [-pal] \| force palindromes (requires -dna); \| [-maxiter <maxiter>] \| maximum EM iterations to run; \| [-distance <distance>] \| EM convergence criterion; \| [-psp <pspfile>] \| name of positional priors file; \| [-prior dirichlet\|dmix\| \| type of prior to use; \|   mega\|megap\|addone] \|; \| [-b <b>] \| strength of the prior; \| [-plib <plib>] \| name of Dirichlet prior file; \| [-spfuzz <spfuzz>] \| fuzziness of sequence to theta mapping; \| [-spmap uni\|pam] \| starting point seq to theta mapping type; \| [-cons <cons>] \| consensus sequence to start EM from; \| [-heapsize <hs>] \| size of heaps for widths where substring; \| \| search occurs; \| [-x\_branch] \| perform x-branching; \| [-w\_branch] \| perform width branching; \| [-bfactor <bf>] \| branching factor for branching search; \| [-maxsize <maxsize>] \| maximum dataset size in characters; \| [-nostatus] \| do not print progress reports to terminal; \| [-p <np>] \| use parallel version with <np> processors; \| [-time <t>] \| quit before <t> CPU seconds consumed; \| [-sf <sf>] \| print <sf> as name of sequence file; \| [-V] \| verbose mode |

MEME is a tool for discovering motifs in a group of related DNA or protein sequences.

A motif is a sequence pattern that occurs repeatedly in a group of related
protein or DNA sequences. MEME represents motifs as position-dependent
letter-probability matrices which describe the probability of each possible
letter at each position in the pattern. Individual MEME motifs do not
contain gaps. Patterns with variable-length gaps are split by MEME into two
or more separate motifs.

MEME takes as input a group of DNA or protein sequences (the training set)
and outputs as many motifs as requested. MEME uses statistical modeling
techniques to automatically choose the best width, number of occurrences,
and description for each motif.

MEME outputs its results primarily as a hypertext (HTML) document
named `meme.html`. This is placed in a directory named `meme_out/`".
You can select for the directory to have a different name.
MEME also outputs machine-readable (XML) a plain-text versions
of its output, named `meme.xml` and `meme.txt`, respectively. These
are placed in the same output directory as `meme.html`.

The MEME results consist of:

- The version of MEME and the date it was released.
- The reference to cite if you use MEME in your research.
- A description of the sequences you submitted (the "training set")
  showing the name, "weight" and length of each sequence.
- The command line summary detailing the parameters with which you
  ran MEME.
- Information on each of the motifs MEME discovered, including:
  1. A summary line showing the width, number of occurrences, log
     likelihood ratio and statistical significance of the motif.
  2. A sequence "LOGO" illustrating the motif, along with links to
     publication-ready versions of the LOGO (postscript and PNG formats).
  3. The occurrences of the motif sorted by *p*-value and aligned with each other.
  4. Block diagrams of the occurrences of the motif within each sequence in the training set.
  5. The motif in BLOCKS format: buttons for viewing the motif in various formats
     and for submitting it to the BLOCKS multiple alignment processor.
  6. A position-specific scoring matrix (PSSM) for use in scanning sequence databases
     and a button for submitting the motif to the MAST scanning program.
  7. The position specific probability matrix (PSPM) describing the motif
     and a button for comparing the motif to known motifs.
  8. A regular expression describing the motif.
- A summary of motifs showing an optimized (non-overlapping) tiling of
  all of the motifs onto each of the sequences in the training set.
- The reason why MEME stopped and the name of the CPU on which it ran.
- This explanation of how to interpret MEME results.

## REQUIRED ARGUMENTS:

- `<dataset>` The name of the file containing the training set sequences.
  If `<dataset>` is the word `stdin`, MEME reads from standard input.

  The sequences in the dataset should be in Pearson/FASTA format. For example:

  ```
            >ICYA_MANSE INSECTICYANIN A FORM (BLUE BILIPROTEIN)
            GDIFYPGYCPDVKPVNDFDLSAFAGAWHEIAK
            LPLENENQGKCTIAEYKYDGKKASVYNSFVSNGVKEYMEGDLEIAPDA
            >LACB_BOVIN BETA-LACTOGLOBULIN PRECURSOR (BETA-LG) 
            MKCLLLALALTCGAQALIVTQTMKGLDI
            QKVAGTWYSLAMAASDISLLDAQSAPLRVYVEELKPTPEGDLEILLQKW
  ```

  Sequences start with a header line followed by
  sequence lines. A header line has
  the character "`>`" in position one, followed by
  an unique name without any spaces, followed by
  (optional) descriptive text. After the header line
  come the actual sequence lines. Spaces and blank
  lines are ignored. Sequences may be in capital or
  lowercase or both.

  MEME uses the first word in the header line of each
  sequence, truncated to 24 characters if necessary,
  as the name of the sequence. This name must be unique.
  Sequences with duplicate names will be ignored.
  (The first word in the title line is
  everything following the "`>`" up to the first blank.)

  Sequence weights may be specified in the dataset
  file by special header lines where the unique name
  is "`WEIGHTS`" (all caps) and the descriptive
  text is a list of sequence weights.
  Sequence weights are numbers in the range `0 < w <= 1`.
  All weights are assigned in order to the
  sequences in the file. If there are more sequences
  than weights, the remainder are given weight one.
  Weights must be greater than zero and less than
  or equal to one. Weights may be specified by
  more than one "`WEIGHT`" entry which may appear
  anywhere in the file. When weights are used,
  sequences will contribute to motifs in proportion
  to their weights. Here is an example for a file
  of three sequences where the first two sequences are
  very similar and it is desired to down-weight them:

  ```
            >WEIGHTS 0.5 .5 1.0 
            >seq1
            GDIFYPGYCPDVKPVNDFDLSAFAGAWHEIAK
            >seq2
            GDMFCPGYCPDVKPVGDFDLSAFAGAWHELAK
            >seq3
            QKVAGTWYSLAMAASDISLLDAQSAPLRVYVEELKPTPEGDLEILLQKW
  ```

## OPTIONAL ARGUMENTS:

MEME has a large number of optional inputs that can be used
to fine-tune its behavior. To make these easier to understand
they are divided into the following categories:

- OUTPUT DESTINATION — control where MEME places its output
- ALPHABET — control the alphabet for the motifs (patterns) that MEME will search for
- DISTRIBUTION — control how MEME assumes the occurrences of the motifs are distributed
  throughout the training set sequences
- SEARCH — control how MEME searches for motifs
- SYSTEM —
  the `-p <np>` argument causes a version of MEME compiled for a parallel CPU
  architecture to be run.
  (By placing `<np>` in quotes you may pass installation specific switches to
  the 'mpirun' command. The number of processors to run on must be the first
  argument following `-p`).

In what follows, `<n>` is an integer, `<a>`
is a decimal number, and `<string>` is a string of characters.

### OUTPUT DESTINATION

By default MEME writes its results to a directory named `meme_out/`,
which is created if it doesn't exist.
The main results include file is `meme.html`.
You can specify that a different directory be created or used for results.
You can also specify that MEME create only a text file.

- `-o <output dir>` — Name of directory for output files;
  will **not** replace existing directory.
- `-oc <output dir>` — Name of directory for output files;
  **will** replace existing directory.
- `-text` — Output in text format only to standard output.

### ALPHABET

MEME accepts either DNA or protein sequences, but not both in the same run.
By default, sequences are assumed to be protein. The sequences must be in
FASTA format.

DNA sequences must contain only the letters "`ACGT`", plus the ambiguous
letters "`BDHKMNRSUVWY*-`".
Protein sequences must contain only the letters "`ACDEFGHIKLMNPQRSTVWY`",
plus the ambiguous letters "`BUXZ*-`".

MEME converts all ambiguous letters to "`X`", which is treated as "unknown".

- `-dna` — Assume sequences are DNA; default: protein sequences.
- `-protein` — Assume sequences are protein.

### DISTRIBUTION

If you know how occurrences of motifs are distributed in the training set
sequences, you can specify it with the following optional switches. The
default distribution of motif occurrences is assumed to be zero or one
occurrence of per sequence.

If you know how occurrences of motifs are distributed in the training set

- `-mod <string>` — The type of distribution to assume.
  - `oops` — One Occurrence Per Sequence MEME assumes that each sequence in the dataset
    contains exactly one occurrence of each motif.
    This option is the fastest and most sensitive but the motifs returned by MEME may be
    "blurry" if any of the sequences is missing them.
  - `zoops` — Zero or One Occurrence Per Sequence
    MEME assumes that each sequence may contain at
    most one occurrence of each motif. This option
    is useful when you suspect that some motifs
    may be missing from some of the sequences. In
    that case, the motifs found will be more
    accurate than using the first option. This
    option takes more computer time than the
    first option (about twice as much) and is
    slightly less sensitive to weak motifs present
    in all of the sequences.
  - `anr` — Any Number of Repetitions
    MEME assumes each sequence may contain any
    number of non-overlapping occurrences of each
    motif. This option is useful when you suspect
    that motifs repeat multiple times within a
    single sequence. In that case, the motifs
    found will be much more accurate than using
    one of the other options. This option can also
    be used to discover repeats within a single
    sequence. This option takes the much more
    computer time than the first option (about ten
    times as much) and is somewhat less sensitive
    to weak motifs which do not repeat within a
    single sequence than the other two options.

### SEARCH

1. OBJECTIVE FUNCTION

   MEME uses an objective function on motifs to select the "best" motif.
   The objective function is based on the statistical significance of the
   log likelihood ratio (LLR) of the occurrences of the motif.
   The *E*-value of the motif is an estimate of the number of motifs (with the
   same width and number of occurrences) that would have equal or higher log
   likelihood ratio if the training set sequences had been generated randomly
   according to the (0-order portion of the) background model.

   MEME searches for the motif with the smallest *E*-value.
   It searches over different motif widths, numbers of occurrences, and
   positions in the training set for the motif occurrences.
   The user may limit the range of motif widths and number of occurrences
   that MEME tries using the switches described below. In addition,
   MEME trims the motif (using a dynamic programming multiple alignment) to
   eliminate any positions where there is a gap in any of the occurrences.

   The log likelihood ratio of a motif is
   `llr = log (Pr(sites | motif) / Pr(sites | back))`
   and is a measure of how different the sites are from the background model.
   Pr(sites | motif) is the probability of the occurrences given the a model
   consisting of the position-specific probability matrix (PSPM) of the motif.
   (The PSPM is output by MEME).
   Pr(sites | back) is the probability of the occurrences given the background
   model. The background model is an n-order Markov model. By default,
   it is a 0-order model consisting of the frequencies of the letters in
   the training set. A different 0-order Markov model or higher order Markov
   models can be specified to MEME using the -bfile option described below.

   The *E*-value reported by MEME is actually an approximation of the *E*-value
   of the log likelihood ratio. (An approximation is used because it is far
   more efficient to compute.) The approximation is based on the fact that
   the log likelihood ratio of a motif is the sum of the log
   likelihood ratios of each column of the motif. Instead of computing the
   statistical significance of this sum (its *p*-value), MEME computes the
   *p*-value of each column and then computes the significance of their product.
   Although not identical to the significance of the log likelihood ratio, this
   easier to compute objective function works very similarly in practice.

   The motif significance is reported as the *E*-value of the motif.
   The statistical significance of a motif is computed based on:

   1. the log likelihood ratio,
   2. the width of the motif,
   3. the number of occurrences,
   4. the 0-order portion of the background model,
   5. the size of the training set, and
   6. the type of model (oops, zoops, or anr, which determines the
      number of possible different motifs of the given width and
      number of occurrences).

   MEME searches for motifs by performing Expectation Maximization (EM) on a
   motif model of a fixed width and using an initial estimate of the number of
   sites. It then sorts the possible sites according to their probability
   according to EM. MEME then and calculates the *E*-values of the first n sites
   in the sorted list for different values of n. This procedure (first EM,
   followed by computing *E*-values for different numbers of sites) is repeated
   with different widths and different initial estimates of the number of
   sites. MEME outputs the motif with the lowest *E*-value.
  
2. NUMBER OF MOTIFS
   - `-nmotifs <n>` — The number of \*different\* motifs to search for.
     MEME will search for and output `<n>` motifs. Default: 1
   - `-evt <p>` — Quit looking for motifs if *E*-value exceeds
     `<p>`.
     Default: infinite (so by default MEME never quits
     before `-nmotifs <n>` have been found.)
  
3. NUMBER OF MOTIF OCCURRENCES
   - `-nsites <n>`   
     `-minsites <n>`   
     `-maxsites <n>`   
     The (expected) number of occurrences of each motif.
     If `-nsites` is given, only that number of occurrences
     is tried. Otherwise, numbers of occurrences between
     `-minsites` and `-maxsites` are tried as initial guesses
     for the number of motif occurrences. These switches are ignored if mod = oops.
       
     Defaults:

     ```
                  -minsites : 2
                  -maxsites :
                    zoops : number of sequences
                    anr   : MIN(5*(number of sequences), 50)
     ```
   - `-wnsites <n>` — The weight on the prior on nsites. This controls
     how strong the bias towards motifs with exactly
     nsites sites (or between minsites and maxsites sites)
     is. It is a number in the range [0..1). The
     larger it is, the stronger the bias towards
     motifs with exactly nsites occurrences is.
       
     Default: 0.8
  
4. MOTIF WIDTH
   - `-w <n>`  
     `-minw <n>`  
     `-maxw <n>`  

     The width of the motif(s) to search for.
     If -w is given, only that width is tried.
     Otherwise, widths between -minw and -maxw are tried.
       
     Default: -minw 8, -maxw 50 (defined in user.h)

     Note: If <n> is less than the length of the shortest
     sequence in the dataset, <n> is reset by MEME to that value.
   - `-nomatrim`   
     `-wg <a>`   
     `-ws <a>`   
     `-noendgaps`   

     These switches control trimming (shortening) of
     motifs using the multiple alignment method.
     Specifying -nomatrim causes MEME to skip this and
     causes the other switches to be ignored.
     MEME finds the best motif
     found and then trims (shortens) it using the multiple
     alignment method (described below). The number of
     occurrences is then adjusted to maximize the motif
     *E*-value, and then the motif width is further
     shortened to optimize the *E*-value.

     The multiple alignment method performs a separate
     pairwise alignment of the site with the highest
     probability and each other possible site.
     (The alignment includes width/2 positions on either
     side of the sites.) The pairwise alignment
     is controlled by the switches:
       
     `-wg <a>` (gap cost; default: 11),   
     `-ws <a>` (space cost; default 1), and,   
     `-noendgaps` (do not penalize endgaps; default:
     penalize endgaps).

     The pairwise alignments are then combined and the
     method determines the widest section of the motif with
     no insertions or deletions.
     If this alignment is shorter than `<minw>`,
     it tries to find an alignment
     allowing up to one insertion/deletion per motif
     column. This continues (allowing up to 2, 3 ...
     insertions/deletions per motif column) until an
     alignment of width at least `<minw>` is found.
5. BACKGROUND MODEL
   - `-bfile <bfile>` — The name of the file containing the background model
     for sequences. The background model is the model
     of random sequences used by MEME. The background
     model is used by MEME
     1. during EM as the "null model",
     2. for calculating the log likelihood ratio of a motif,
     3. for calculating the significance (*E*-value) of a motif, and,
     4. for creating the position-specific scoring matrix (log-odds matrix).

   By default, the background model is a 0-order Markov
   model based on the letter frequencies in the training set.

   Markov models of any order can be specified in <bfile>
   by listing frequencies of all possible tuples of
   length up to order+1.

   Note that MEME uses only the 0-order portion (single
   letter frequencies) of the background model for
   purposes 3) and 4), but uses the full-order model
   for purposes 1) and 2), above.

   Example: To specify a 1-order Markov background model
   for DNA, `<bfile>` might contain the following
   lines. Note that optional comment lines are
   marked by "#" and are ignored by MEME.

   ```
                   # tuple   frequency_non_coding
                   a       0.324
                   c       0.176
                   g       0.176
                   t       0.324
                   # tuple   frequency_non_coding
                   aa      0.119
                   ac      0.052
                   ag      0.056
                   at      0.097
                   ca      0.058
                   cc      0.033
                   cg      0.028
                   ct      0.056
                   ga      0.056
                   gc      0.035
                   gg      0.033
                   gt      0.052
                   ta      0.091
                   tc      0.056
                   tg      0.058
                   tt      0.119
   ```

   Sample -bfile files are given in directory tests:
   `tests/nt.freq` (DNA), and
   `tests/na.freq` (amino acid).
6. POSITION-SPECIFIC PRIORS
   - `-psp <pspfile>` —
     position-specific prior (PSP)

     These priors allow the user to bias the search for motifs.
     They give a position-specific prior distribution on the location of motif sites
     in sequence(s) in the input dataset. The MEME PSP format used in the pspfile
     includes the name of the sequence for which a prior distribution corresponds.
     Sequences not named in the pspfile are given uniform prior distributions on
     site locations by MEME.

     A PSP must be created for a specific width of motif, `w`.
     This width must be specified for each entry in the pspfile, and must be the same
     for all entries. If MEME varies the motif width during computation,
     MEME renormalises the PSP for each sequence.

     The pspfile should be in MEME PSP format, which is similar to FASTA format. For example:

     ```
         >ICYA_MANSE 4
         0.075922 0.070764 0.082380 0.030292 0.025101 0.043139 0.032963
         0.086047 0.057445 0.000000 0.000000 0.000000

         >LACB_BOVIN 4
         0.107099 0.099822 0.116208 0.042731 0.035408 0.060854 0.046499
         0.000000 0.000000 0.000000
     ```

     Each entry should start with a header line consisting of a sequence
     name followed by the width, `w`, of the PSP prior. The sequence name must
     the name of a sequence in the FASTA file input to MEME. Any other
     text on the header line after the name and `w` is
     ignored by MEME.
     The following lines contain one number for each position in the identically-named
     named FASTA sequence, where the number gives the prior probability of a motif
     site at that position in the sequence (or in the reverse complement if
     `-revcomp` is specified).
     The last `w-1` numbers for each entry should be
     0 (shown in blue in the example), since a motif of that width cannot start in those positions.
     All numbers for an entry must be in the range [0,1], and must sum to a number no greater
     than 1. If they sum to less than 1 and `-mod oops` is specified,
     MEME will rescale the numbers so that they sum to 1.

     For more detail on generation of PSP data, see documentation
     of our simple tool psp-gen.
7. DNA PALINDROMES AND STRANDS
   - `-revcomp` — motifs occurrences may be on the given DNA strand
     or on its reverse complement.
       
     Default: look for DNA motifs only on the strand given
     in the training set.
   - `-pal` — Choosing -pal causes MEME to look for palindromes in DNA datasets.

   MEME averages the letter frequencies in corresponding
   columns of the motif (PSPM) together. For instance,
   if the width of the motif is 10, columns 1 and 10, 2
   and 9, 3 and 8, etc., are averaged together. The
   averaging combines the frequency of A in one column
   with T in the other, and the frequency of C in one
   column with G in the other.
   If neither option is not chosen, MEME does not
   search for DNA palindromes.
8. EM ALGORITHM
   - `-maxiter <n>` — The number of iterations of EM to run from
     any starting point. EM is run for `<n>` iterations or until convergence
     (see `-distance`, below) from each starting point.
     Default: 50
   - `-distance <a>` — The convergence criterion.
     MEME stops iterating EM when the change in the
     motif frequency matrix is less than <a>
     (Change is the euclidean distance between
     two successive frequency matrices.)
     Default: 0.001
   - `-prior <string>` — The prior distribution on the model parameters:
     - `dirichlet` — simple Dirichlet prior.
       This is the default for `-dna`.
       It is based on the non-redundant database letter frequencies.
     - `dmix` — mixture of Dirichlets prior.
       This is the default for `-protein`.
     - `mega` — extremely low variance dmix; variance is scaled
       inversely with the size of the dataset.
     - `megap` — `mega` for all but last iteration of EM;
       `dmix`
       on last iteration.
     - `addone` — add +1 to each observed count
   - `-b <a>` — The strength of the prior on model parameters:
     `<a> = 0` means use intrinsic strength of prior for
     `prior = dmix`.
       
     Defaults:
       
     - 0.01 — if prior = dirichlet
     - 0 — if prior = dmix
   - `-plib <string>` — The name of the file containing the Dirichlet prior
     in the format of file prior30.plib.
  
9. SELECTING STARTS FOR EM

   The default is for MEME to search the dataset for good starts for EM. How
   the starting points are derived from the dataset is specified by the
   following switches.

   The default type of mapping MEME uses is:

   - `-spmap uni` — for `-dna`
   - `-spmap pam` — for `-protein`
   - `-spfuzz <a>` — The fuzziness of the mapping.
     Possible values are greater than 0. Meaning
     depends on `-spmap`, see below.
   - `-spmap <string>` — The type of mapping function to use.
     - `uni` — Use add-<a> prior when converting a
       substring to an estimate of theta.
         
       Default `-spfuzz <a>` 0.5
     - `pam` — Use columns of PAM <a> matrix when
       converting a substring to an estimate of theta.
         
       Default `-spfuzz <a>` 120 (PAM 120)

     Other types of starting points
     can be specified using the following switches.
   - `-cons <string>` — Override the sampling of starting points
     and just use a starting point derived from
     `<string>`.
     This is useful when an actual occurrence of
     a motif is known and can be used as the
     starting point for finding the motif.
  
10. BRANCHING SEARCH ON EM STARTS

    The search for good EM starting points can be improved by using branching
    search.

    Branching search begins with a fixed-sized heap of best EM starts identified
    during the search of subsequences from the dataset. These starts are also
    called "seeds". The fixed-sized heap of seeds is used as the "branch\_heap"
    during the first iteration of branching search.

    For each iteration of branching search, all seeds in the current branch\_heap
    are considered. All seeds in the ball within hamming distance 1 of a given
    seed are evaluated and added to a new heap. The ball of new seeds is generated
    by mutating each character of the initial seed to each alternative character
    in the alphabet.

    After the ball for every branch\_heap seed has been evaluated, the seeds in the
    resulting new heap are added to the heap of best EM starts. The new heap is
    then used as the branch\_heap for the next iteration of branching search.

    By default MEME does not perform x-branching.

    - `-x_branch` — Perform x-branching.
    - `-bfactor <bf>` — The number of iterations of branching search. The
      default number of branching iterations is three.
    - `-heapsize <hs>` — The maximum size of the heaps used during branching
      search. The default heap size is 64.

## EXAMPLES:

The following examples use data files provided in this release of MEME.
MEME writes its output to standard output, so you will want to redirect it
to a file in order for use with MAST.

1. A simple DNA example:

   ```
         meme crp0.s -dna -mod oops -pal
   ```

   MEME looks for a single motif in the file `crp0.s` which contains DNA
   sequences in FASTA format. The OOPS model is used so MEME assumes that
   every sequence contains exactly one occurrence of the motif. The
   palindrome switch is given so the motif model (PSPM) is converted into a
   palindrome by combining corresponding frequency columns. MEME automatically
   chooses the best width for the motif in this example since no width was
   specified.
2. Searching for motifs on both DNA strands:

   ```
         meme crp0.s -dna -mod oops -revcomp
   ```

   This is like the previous example except that the `-revcomp` switch tells
   MEME to consider both DNA strands, and the `-pal` switch is absent so the
   palindrome conversion is omitted. When DNA uses both DNA strands, motif
   occurrences on the two strands may not overlap. That is, any position
   in the sequence given in the training set may be contained in an occurrence
   of a motif on the positive strand or the negative strand, but not both.
3. A fast DNA example:

   ```
         meme crp0.s -dna -mod oops -revcomp -w 20
   ```

   This example differs from example 1) in that MEME is told to only
   consider motifs of width 20. This causes MEME to execute about 10
   times faster. The `-w` switch can also be used with protein datasets if
   the width of the motifs is known in advance.
4. Using a higher-order background model:

   ```
         meme INO_up800.s -dna -mod anr -revcomp -bfile yeast.nc.6.freq
   ```

   In this example we use `-mod anr` and `-bfile yeast.nc.6.freq`.
   This specifies that
     
   a) the motif may have any number of occurrences in each sequence, and,
     
   b) the Markov model specified in yeast.nc.6.freq is used as the
   background model. This file contains a fifth-order Markov model
   for the non-coding regions in the yeast genome.

   Using a higher order background model can often result in more sensitive
   detection of motifs. This is because the background model more accurately
   models non-motif sequence, allowing MEME to discriminate against it and find
   the true motifs.
5. A simple protein example:

   ```
         meme lipocalin.s -mod oops -maxw 20 -nmotifs 2
   ```

   The `-dna` switch is absent, so MEME assumes the file
   `lipocalin.s` contains protein sequences.
   MEME searches for two motifs each of width less than or equal to 20.
   (Specifying `-maxw 20` makes MEME run faster since it does not have to
   consider motifs longer than 20.) Each motif is assumed to occur in each
   of the sequences because the OOPS model is specified.
6. Another simple protein example:

   ```
         meme farntrans5.s -mod anr -maxw 40 -maxsites 50
   ```

   MEME searches for a motif of width up to 40 with up to 50 occurrences in
   the entire training set. The ANR sequence model is specified,
   which allows each motif to have any number of occurrences in each sequence.
   This dataset contains motifs with multiple repeats of motifs in each
   sequence. This example is fairly time consuming due to the fact that the
   time required to initial the motif probability tables is proportional
   to `<maxw>` times `<maxsites>`.
   By default, MEME only looks for motifs up to
   29 letters wide with a maximum total of number of occurrences equal to twice
   the number of sequences or 30, whichever is less.
7. A much faster protein example:

   ```
         meme farntrans5.s -mod anr -w 10 -maxsites 30 -nmotifs 3
   ```

   This time MEME is constrained to search for three motifs of width exactly ten.
   The effect is to break up the long motif found in the previous example.
   The `-w` switch forces motifs to be \*exactly\* ten letters wide.
   This example is much faster because, since only one width is considered,
   the time to build the motif probability tables is only proportional to
   `<maxsites>`.
8. Splitting the sites into three:

   ```
         meme farntrans5.s -mod anr -maxw 12 -nsites 24 -nmotifs 3
   ```

   This forces each motif to have 24 occurrences, exactly, and be up to 12 letters wide.
9. A larger protein example with *E*-value cutoff:

   ```
         meme adh.s -mod zoops -nmotifs 20 -evt 0.01
   ```

   In this example, MEME looks for up to 20 motifs, but stops when a motif is
   found with *E*-value greater than 0.01. Motifs with large *E*-values are likely
   to be statistical artifacts rather than biologically significant.
